# Association of Epithelial Alarmins and Th2 Cytokines with Disease Severity in Non-Cystic Fibrosis Bronchiectasis

**DOI:** 10.3390/medicina62101852

**Published:** 2026-09-24

**Authors:** Okan Gurbuz, Oya Baydar Toprak, Gulcin Daglioglu, Efraim Guzel, Burak Mete, Bengu Curuk, Ezgi Ozyilmaz

**Affiliations:** 1 Department of Chest Disease, Gaziantep City Hospital, Gaziantep 27470, Turkiye; 2 Department of Chest Disease, Cukurova University, Adana 01330, Turkiye; efraimguzel@gmail.com (E.G.);; 3 Department of Biochemistry, Cukurova University, Adana 01330, Turkiye; 4 Department of Public Health, Cukurova University, Adana 01330, Turkiye

**Keywords:** bronchiectasis, alarmins, IL-25, Th2 inflammation, disease severity, exacerbation, FACED, BSI

## Abstract

*Background and Objectives*: Alarmins and Th2-driven inflammatory pathways may contribute to disease severity, exacerbation frequency, and mortality; however, their role in non-cystic fibrosis bronchiectasis remains insufficiently defined. This study aimed to investigate the association between alarmins, Th2 inflammation, disease severity, exacerbations, and mortality in patients with non-cystic fibrosis bronchiectasis. *Materials and Methods*: A total of 79 adult patients with stable state non-cystic fibrosis bronchiectasis were prospectively enrolled. Demographic characteristics, comorbidities, pulmonary function tests, radiological findings, and disease severity scores (Bronchiectasis Severity Index [BSI] and FACED score [FEV1, age, chronic colonization, extension, dyspnea]) were recorded. Serum levels of IL-25, IL-33, thymic stromal lymphopoietin (TSLP), IL-4, IL-5, IL-13, and IL-17A were measured using enzyme-linked immunosorbent assay. Associations between cytokine levels, disease severity, exacerbations, hospitalizations, and mortality were analyzed using correlation and logistic regression analyses. *Results*: The mean age of the patients was 50.19 years (range: 19–80). Etiological analysis revealed that 40.5% of cases were post-infectious, 22.8% were related to COPD, and 34.2% were associated with asthma. Pulmonary function tests showed obstructive patterns in 38% of patients, while 32.9% had normal findings. Increased IL-25 and IL-5 levels were associated with higher exacerbation frequency, emergency department visits, and hospitalizations. IL-25 levels showed significant correlations with IL-33 and TSLP. According to FACED classification, disease severity increased in parallel with IL-25 and IL-5 levels, whereas no significant association was observed between BSI scores and cytokine levels. Logistic regression analysis identified elevated IL-25 levels, recent hospitalizations, and emergency department admissions as independent risk factors for mortality and morbidity at 1-year follow-up. Agreement between FACED and BSI scores was modest (κ = 0.273). *Conclusions*: Alarmins, particularly IL-25, and Th2-driven inflammation may have a significant association with disease severity and clinical outcomes in non-cystic fibrosis bronchiectasis. Elevated IL-25 and IL-5 levels may serve as clinically relevant biomarkers. Combined use of FACED and BSI scores may improve risk stratification, and targeting alarmin-mediated inflammatory pathways could offer novel therapeutic opportunities.

## 1. Introduction

Bronchiectasis is a progressive lung disease characterized by recurrent infections, chronic inflammation, and irreversible bronchial wall dilatation. It is a heterogeneous respiratory disorder associated with impaired quality of life and progressive lung damage [1].

Chronic inflammation represents a central component of bronchiectasis pathophysiology. Although the disease has traditionally been regarded as predominantly neutrophil-driven [1], recent studies have demonstrated that eosinophilic inflammation also plays a role in approximately one-third of patients with bronchiectasis [2].

Type 2 inflammation is initiated when airway epithelial cells are exposed to allergic or non-allergic stimuli. Following this exposure, epithelial-derived alarmins, including IL-25, IL-33, and thymic stromal lymphopoietin (TSLP), are released. These alarmins activate various immune cell populations, triggering the secretion of type 2 cytokines such as IL-4, IL-5, and IL-13 and initiating a type 2 immune response [3]. Although bronchiectasis involves type 2 pathways, disease progression is primarily driven by infection; therefore, we evaluated IL-17A levels to assess the impact of neutrophilic inflammation. This process results in pathological changes including mucus hypersecretion, airway smooth muscle contraction, and fibrotic remodeling [4,5].

Initiation of immunological mechanisms requires contact of airway epithelial cells with allergic or non-allergic stimuli, such as cigarette smoke, air pollution, and viral infections [6]. Following exposure, alarmins (TSLP, IL-25, and IL-33) which trigger a robust type 2 immune response by acting dendritic cells, basophils, type 2 innate cells (ILC2s) and eosinophils [4,5].

In patients with bronchiectasis, eosinophilic inflammation—observed in nearly one-third of cases—has been associated with elevated fractional exhaled nitric oxide (FeNO) levels, peripheral eosinophilia, IL-13–related type 2 inflammation, and impaired lung function [7].

Alarmins are potent cytokines that strongly activate ILC2s by engaging MAPK and NF-κB signaling pathways, leading to rapid proliferation and enhanced cytokine secretion. IL-33, a member of the IL-1 cytokine family, is released following cellular injury and increases cytokine production through p38 MAPK–mediated GATA3 phosphorylation [8]. IL-25 belongs to the IL-17 cytokine family and is secreted by epithelial and tracheal cells. During allergen stimulation and inflammation, IL-25 is also produced by Th2 cells, mast cells, alveolar macrophages, basophils, and eosinophils [9,10,11,12]. TSLP is an epithelial-derived cytokine that was first identified in thymic stromal cell cultures [13]. By binding to the TSLP receptor and IL-7 receptor α chain, TSLP activates the JAK1–JAK2 signaling pathway, leading to STAT5 activation. This signaling cascade promotes B-cell development, enhances dendritic cell survival, maturation, and cytokine production, drives Th2 differentiation and regulatory T-cell development, and activates mast cells, eosinophils, and macrophages [14,15]. Alarmins stimulate dendritic cells to express OX40 ligand and secrete type 2 cytokines [4,16]. Dendritic cells, basophils, and ILC2s release IL-4, promoting the differentiation of naïve Th0 cells into Th2 cells. IL-4 and IL-13 secreted by Th2 cells stimulate IgE production by B lymphocytes, while IgE subsequently binds to its specific epitopes on mast cells and basophils. IL-5 produced by Th2 cells enhances eosinophil maturation, proliferation, and survival [4].

As a result, eosinophils, basophils, and mast cells activated by IL-4, IL-5, and IL-13 release histamine, prostaglandins, and leukotrienes, leading to bronchoconstriction, mucus production, mucosal edema, airway smooth muscle contraction, and fibrosis [5,17]. Recent multicenter studies have demonstrated that the incidence of eosinophilic inflammation in bronchiectasis patients is 22.6% [18].

Therapeutic options for bronchiectasis remain limited. Therefore, elucidating the inflammatory pathways underlying the disease and their relationship with disease severity is of critical importance for identifying potential novel therapeutic targets. Although the pathogenesis of type 2 inflammation is well-established in the current literature, disease progression in bronchiectasis is frequently attributed to recurrent infections, impaired mucociliary clearance, and chronic colonization. However, as recent studies demonstrate a high prevalence of eosinophilic type 2 inflammation among these patients, this study aimed to investigate its relationship with overall disease severity.

In this study, we aimed to investigate the relationship between type 2 inflammation, epithelial alarmins, and disease severity in patients with non-cystic fibrosis bronchiectasis. To identify type 2 inflammation in bronchiectasis, the current literature indicates that a peripheral blood eosinophil count of ≥150 cells/µL serves as an adequate clinical indicator [19]. Specifically, we sought to evaluate the effects of these inflammatory biomarkers on exacerbation risk and overall disease severity. For this purpose, the Bronchiectasis Severity Index (BSI) and the FACED score were utilized to correlate the inflammatory burden with clinical severity. These scoring systems were chosen to capture complementary clinical dimensions: the BSI was specifically selected for its ability to predict future exacerbations, while the FACED score was employed due to its established success in predicting mortality. Ultimately, these analyses aim to elucidate the specific contribution of type 2 inflammation to bronchiectasis progression and provide a deeper understanding of its underlying pathogenesis.

## 2. Materials and Methods

### 2.1. Study Design and Study Population

This prospective observational study was conducted between October 2024 and October 2025 and included adult patients diagnosed with non-cystic fibrosis bronchiectasis who presented to the Chest Diseases Outpatient Clinic of Çukurova University Balcalı Hospital. All participants provided written informed consent prior to enrollment, in accordance with the Declaration of Helsinki.

Inclusion criteria were as follows:

- age ≥ 18 years;
- willingness to participate and provision of written informed consent signed;
- diagnosis of bronchiectasis confirmed by high-resolution computed tomography (HRCT) in conjunction with clinical history and physical examination. Eligible patients were enrolled consecutively to minimize selection bias.

Exclusion criteria included:

- refusal or inability to provide informed consent;
- inability to perform acceptable and reproducible pulmonary function tests;
- bronchiectasis secondary to cystic fibrosis.

For all enrolled patients, sociodemographic characteristics, detailed medical history, and physical examination findings were recorded using a standardized data collection form. Pre- bronchodilator pulmonary function tests were performed for all patients, and HRCT was obtained when not previously available. Etiological evaluation of bronchiectasis was conducted by chest disease specialists in accordance with current international guidelines, using a standardized diagnostic work-up to ensure reproducibility across patients.

Patient were excluded from the study if they experienced an exacerbation, defined as clinical deterioration lasting for at least 48 h (increased cough severity/frequency, increased sputum volume/consistency, increased sputum purulence, increased dyspnea/reduced exercise tolerance, malaise and hemoptysis).

### 2.2. Pulmonary Function Tests

Pulmonary function tests (PFTs) were performed in the Pulmonary Function Testing Laboratory of the Chest Diseases Department using a calibrated Sensor Medics V-Max 20 spirometer (Jaeger MS-PFT Analyzer Unit, Vyaire Medical GmbH, Höchberg, Germany). All measurements were conducted in accordance with ATS/ERS standards18.

Forced expiratory volume in one second (FEV_1_) and forced vital capacity (FVC) were measured three times, and the best reproducible measurement was recorded for analysis. The following spirometric parameters were evaluated: FEV_1_ (L and % predicted), FVC (L and % predicted), FEV_1_/FVC ratio, and forced expiratory flow between 25% and 75% of FVC (FEF_25–75_; L/s and % predicted). This standardized protocol ensured consistency and reproducibility of lung function measurements.

### 2.3. Skin Prick Testing

Skin prick testing was performed by an experienced pulmonary function technician in the Department of Chest Diseases. The allergen panel included Dermatophagoides farinae, Dermatophagoides pteronyssinus, cat, dog, Betulaceae (tree pollens), grass mix, Salicaceae (poplar–willow), and Aspergillus species, using commercial test kits (Starallergens, Paris, France).

To prevent false-negative or false-positive results, medications known to interfere with skin testing—including tricyclic antidepressants, antiemetics, systemic and topical corticosteroids, H_2_-receptor antagonists, antihistamines, and montelukast—were discontinued for an appropriate washout period prior to testing, in accordance with manufacturer recommendations.

Histamine hydrochloride (10 mg/mL) was used as a positive control, and the diluent solution served as a negative control. Test results were evaluated at 20 min using a standardized ruler. A wheal diameter ≥ 4 mm for the positive control and ≤3 mm for the negative control was considered indicative of a valid test. Skin prick test positivity was defined as a wheal diameter greater than the negative control and ≥5 mm, or ≥70% of the positive control response, in line with kit specifications.

### 2.4. FACED and Bronchiectasis Severity Index (BSI)

Disease severity was assessed using both the FACED score and the Bronchiectasis Severity Index (BSI). The FACED score incorporates FEV_1_ (% predicted), age, chronic Pseudomonas aeruginosa colonization, radiological extent (number of affected lobes), and dyspnea severity assessed by the modified Medical Research Council (mMRC) scale, yielding a total score ranging from 0 to 7.19.

The BSI score includes age, body mass index, FEV_1_ (% predicted), prior hospitalizations, number of exacerbations in the previous year, mMRC score, chronic colonization with Pseudomonas aeruginosa or other microorganisms, and radiological severity [20]. Scores range from 0 to ≥26. Patients were categorized as having mild, moderate, or severe disease according to established cut-off values for each scoring system, allowing standardized comparison across severity groups.

### 2.5. Reiff Score

Radiological severity on HRCT was evaluated using the Reiff score, a validated scoring system widely used in bronchiectasis studies. The Reiff score assesses the degree of bronchial dilatation and classifies bronchiectasis as tubular (score 1), varicose (score 2), or cystic (score 3), enabling reproducible assessment of morphological severity [21].

### 2.6. Laboratory Measurements and ELISA Analysis

Peripheral venous blood samples (10 mL) were collected from the antecubital vein into BD Vacutainer^®^ SST™ II Advance gel tubes (Plymount, England). Samples were centrifuged using a Nüve NF 1200 centrifuge (Ankara, Turkiye), and serum aliquots were stored at −80 °C until analysis to preserve biomarker stability.

Serum levels of IL-4 (Catalog NO:RE1055H), IL-5 (Catalog NO:RE1099H), IL-13 (Catalog NO:RE1056H), IL-17A (Catalog NO:RE2867H), IL-25( Catalog NO:RE1958H), IL-33 (Catalog NO:RE2428H), and human thymic stromal lymphopoietin (TSLP) (Catalog NO:RE1929H) were measured using commercial sandwich ELISA kits (Reed Biotech, Wuhan, China) according to the manufacturers’ protocols. Analyses were performed using a CHROMATE semi-automated micro-ELISA analyzer (FL, USA) at the Çukurova University Faculty of Medicine Central Laboratory. The lower limits of detection (LOD) for the ELİSA kits were 31.25 pg/mL for TSLP and 3.13 pg/mL for IL-33. All samples were analyzed under identical conditions, and results were reported in pg/mL to ensure analytical reproducibility.

### 2.7. Statistical Analysis

All statistical analyses were performed using IBM SPSS Statistics for Windows, version 27 (IBM Corp., Armonk, NY, USA). Continuous variables were summarized as mean ± standard deviation or median [interquartile range], while categorical variables were presented as frequencies and percentages. The distribution of continuous variables was assessed for normality using the Shapiro–Wilk test, together with visual inspection of histograms and Q–Q plots. A *p* value > 0.05 in the Shapiro–Wilk test was considered compatible with a normal distribution.

Associations between categorical variables were assessed using Pearson’s chi-square test or the Fisher–Freeman–Halton test, as appropriate. When significant differences were identified, post hoc comparisons with Bonferroni correction were applied. Logistic regression analyses were conducted to identify variables associated with disease severity and mortality. Variables with a *p* value ≤ 0.25 in univariate analysis were entered into multivariable models using backward elimination. The primary multivariable logistic regression models were additionally adjusted for age, gender, and smoking status as clinically relevant potential confounders, irrespective of their statistical significance in univariate analyses. Odds ratios (ORs) and 95% confidence intervals (CIs) were calculated. A two-sided *p* value < 0.05 was considered statistically significant. All analyses followed predefined statistical plans to ensure transparency and reproducibility.

## 3. Results

### 3.1. Sociodemographic and Clinical Characteristics of the Study Population

A total of 79 adult patients with non-cystic fibrosis bronchiectasis were included in the study. Of these, 44 patients (55.7%) were female and 35 (44.3%) were male. The mean age of the study population was 50.19 ± 17.67 years, ranging from 19 to 80 years. The mean body mass index (BMI) was 20.96 ± 2.78 kg/m^2^. A history of smoking was present in 22 patients (27.8%), whereas 57 patients (72.2%) had never smoked.

Comorbid diseases were frequent: Chronic obstructive pulmonary disease (COPD) was present in 18 patients (22.8%), asthma in 27 patients (34.2%), diabetes mellitus in 9 patients (11.4%), hypertension in 18 patients (22.8%), coronary artery disease in 13 patients (16.5%), immunodeficiency in 4 patients (5.1%), rheumatologic diseases in 7 patients (8.9%), psychiatric disorders in 14 patients (17.7%), inflammatory bowel disease in 1 patient (1.3%), and sinusitis in 6 patients (7.6%). Sociodemographic characteristics of the study population are summarized in Table 1.

### 3.2. Etiology, Radiological Features, and Pulmonary Function Tests

The most common etiologies of bronchiectasis were post-infectious causes in 32 patients (40.5%), asthma in 27 patients (34.2%), and COPD in 18 patients (22.8%). Other etiologies included previous tuberculosis (7.6%), allergic bronchopulmonary aspergillosis (3.8%), immunodeficiency (5.1%), rheumatologic diseases (8.9%), primary ciliary dyskinesia (6.3%), inflammatory bowel disease (1.3%), and other rare causes such as Mounier–Kuhn syndrome and Young syndrome (7.6%).

Radiological assessment revealed tubular bronchiectasis in 11 patients (14.5%), varicose bronchiectasis in 11 patients (14.5%), and cystic bronchiectasis in 57 patients (71%). One lobe was affected in 10 patients (12.7%), two lobes in 28 patients (34.5%), three lobes in 15 patients (19%), four lobes in 7 patients (8.9%), and five lobes in 19 patients (24.1%). The most frequently involved lobes were the left upper lobe and lingula (68.4%), left lower lobe (67.1%), right upper lobe (62%), right lower lobe (57%), and right middle lobe (46.8%).

Pulmonary function tests showed a mean FEV1/FVC ratio of 67.94 ± 12.64%. Mean FEV1 was 65.36 ± 24.58% predicted (1.88 ± 0.95 L), and mean FVC was 76.82 ± 23.48% predicted (2.66 ± 1.10 L). Mean FEF25–75 was 37.61 ± 25.09% (2.53 ± 10.35 L). Based on spirometric findings, 26 patients (32.9%) had normal pulmonary function, 30 patients (38%) had obstructive impairment, 9 patients (11.4%) had restrictive impairment, and 9 patients (11.4%) had mixed impairment. Detailed clinical characteristics are presented in Table 2.

### 3.3. Bronchiectasis Severity Index (BSI) Analysis

According to the BSI classification, 31 patients (39.2%) had mild disease, 16 patients (20.3%) had moderate disease, and 32 patients (40.5%) had severe disease. Comparisons among BSI severity groups revealed statistically significant differences in neutrophil count (*p* = 0.042), C-reactive protein (CRP) levels (*p* = 0.004), and CD8+ T-cell counts (*p* = 0.022).

Pulmonary function parameters, including FEV1/FVC, FEV1, FVC, and FEF25–75 (both absolute and percentage values), showed significant decreases with increasing disease severity (*p* < 0.001 for all). Although significant differences were observed in acute-phase reactants such as neutrophil count and CRP levels, no statistically significant difference was found in erythrocyte sedimentation rate (ESR) among BSI groups.

Serum levels of IL-25, IL-33, thymic stromal lymphopoietin (TSLP), IL-4, IL-5, IL-13, and IL-17A did not differ significantly among BSI severity groups Table 3. Correlation analysis revealed a weak but statistically significant positive correlation between BSI score and IL-13 levels (r = 0.212, *p* < 0.05) (Figure 1). No significant correlations were observed between BSI scores and IL-33, TSLP, or IL-17A levels.

**Figure 1:**
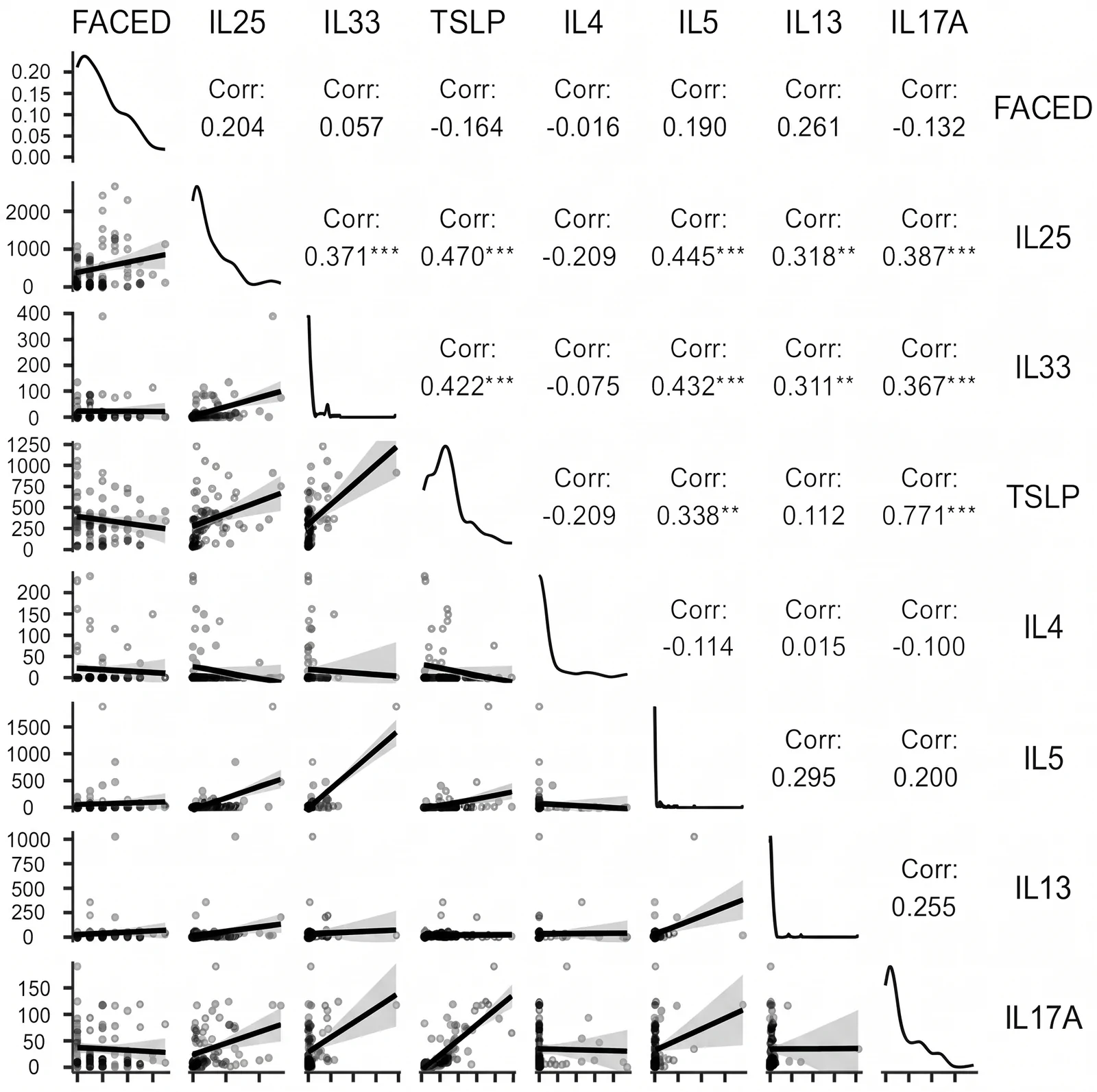
Investigation of the positive correlation between FACED scores and interleukin levels. * *p* < 0.05, ** *p* < 0.01, *** *p* < 0.001.

**Table 3:** Comparison of alarmin levels based on BSI scores.

Interleukin	Disease Severity (BSI)	n	Median [IQR]	*p*-Value
IL-25	Mild	31	250 [597.426] pg/mL	0.822
	Moderate	16	355.95 [1232.921] pg/mL	
	Severe	31	266.66 [738.448] pg/mL	
IL-33	Mild	31	9.23 [58.23] pg/mL	0.497
	Moderate	16	0.57 [16.58] pg/mL	
	Severe	31	4.41 [10.19] pg/mL	
TSLP	Mild	31	360.66 [381.206] pg/mL	0.111
	Moderate	16	289.01 [316.094] pg/mL	
	Severe	31	255.57 [315.479] pg/mL	
IL-4	Mild	31	0 [0] pg/mL	0.606
	Moderate	16	0 [26.78] pg/mL	
	Severe	31	0 [0] pg/mL	
IL-5	Mild	31	0 [25.17] pg/mL	0.819
	Moderate	16	5.1 [19.88] pg/mL	
	Severe	31	8.41 [23.44] pg/mL	
IL-13	Mild	31	9.26 [22.1] pg/mL	0.395
	Moderate	16	10.71 [41.33] pg/mL	
	Severe	31	18.67 [32.22] pg/mL	
IL-17A	Mild	31	29.57 [54.65] pg/mL	0.304
	Moderate	16	12.04 [38.81] pg/mL	
	Severe	31	11.01 [70.06] pg/mL	

IQR: Interquartile Range.

### 3.4. FACED Score Analysis

According to the FACED score, 54 patients (68.4%) were classified as having mild disease, 16 patients (20.3%) moderate disease, and 9 patients (11.4%) severe disease. Significant differences among FACED severity groups were observed for BMI (*p* = 0.013), neutrophil count (*p* = 0.002), and CRP levels (*p* = 0.014).

Pulmonary function parameters (FEV1/FVC, FEV1, FVC, and FEF25–75) decreased significantly with increasing FACED severity (*p* < 0.001 for all).

FACED severity was significantly associated with serum IL-25 (*p* = 0.016) and IL-5 (*p* = 0.031) levels, both of which increased with disease severity. No significant differences were observed for IL-33, TSLP, IL-4, IL-13, or IL-17A levels across FACED groups (Table 4). Correlation analysis demonstrated weak but significant positive correlations between FACED scores and IL-25, IL-5, and IL-13 levels (Table 4, Figure 2).

### 3.5. Agreement Between FACED and BSI Scores

When FACED and BSI classifications were compared, the overall agreement rate was 58%. Cohen’s kappa analysis demonstrated weak agreement between the two scoring systems (κ = 0.273), although the agreement was statistically significant (*p* < 0.001). The cross-tabulation of FACED and BSI scores is presented in Table 5.

### 3.6. Logistic Regression Analyses

In logistic regression analysis predicting moderate-to-severe disease according to FACED score, the final forward stepwise model was statistically significant (omnibus *p* < 0.001). IL-25 levels, history of hospitalization, and emergency department admissions were identified as independent risk factors. Each 1 pg/mL increase in IL-25 level increased the risk of exacerbation by 1.002-fold, while hospitalization within the previous year increased the risk by 5.22-fold and emergency department admission by 2.19-fold (Table 6).

For BSI classification, logistic regression analysis demonstrated that IL-25 levels, emergency department admission, and hospitalization were independent predictors of moderate-to-severe disease (omnibus *p* < 0.001). Each 1 pg/mL increase in IL-25 level increased the risk by 1.003-fold, hospitalization increased the risk by 40.26-fold, and emergency department admission increased the risk by 2.75-fold (Table 7). Notably, IL-25 was the only cytokine consistently associated with both clinical outcomes and disease severity across multiple analyses.

**Table 7:** Logistic regression analysis by BSI score.

BSI	Parameters	B	Standard Error	*p*	OR	95% IR	
						Lower limit	Upper limit
	IL-25	0.003	0.001	**0.014 **	1.003	1.001	1.005
	IL33	−0.033	0.018	0.07	0.967	0.933	1.003
	TSLP	−0.003	0.003	0.303	0.997	0.991	1.003
	IL4	0.006	0.007	0.393	1.006	0.993	1.019
	IL-5	0.001	0.006	0.889	1.001	0.989	1.013
	IL13	−0.005	0.009	0.588	0.995	0.977	1.013
	IL17A	0.001	0.017	0.932	1.001	0.969	1.035
	BMI	0.012	0.173	0.946	1.012	0.721	1.421
	ER admission	1.014	0.404	**0.012 **	2.757	1.249	6.085
	Hospitalization	3.695	1.305	**0.005 **	40.262	3.119	519.763

BMI: Body mass index, ER: Emergency room IR: Confidence İnterval. Bold typeface is used for statistically significant data.

## 4. Discussion

The present study may demonstrate that epithelial alarmins, particularly IL-25, are significantly associated with disease severity, exacerbation burden, and healthcare utilization in patients with non-cystic fibrosis bronchiectasis. IL-25 was the only cytokine consistently associated with both clinical outcomes and severity indices, suggesting a central role in disease pathophysiology. Additionally, its correlation with IL-33 and TSLP supports the presence of an epithelial-driven inflammatory network contributing to disease progression.

When bronchiectasis etiology was evaluated, post-infectious causes were the most frequent, followed by asthma, COPD, previous tuberculosis, rheumatologic diseases, primary ciliary dyskinesia, and other rare causes. Previous studies have reported varying etiological distributions, with post-infectious bronchiectasis reported in up to 67.5%, asthma in 6.3%, COPD in 8.8%, and idiopathic causes in approximately 12.9% of cases [20]. Another cohort study reported idiopathic bronchiectasis in 47.4%, childhood respiratory diseases in 22.7%, post-pneumonic bronchiectasis in 11.5%, and tuberculosis-related disease in 15.5% [21]. In a study conducted in India, tuberculosis remained the dominant cause (50%), followed by post-pneumonic bronchiectasis (36.3%), allergic bronchopulmonary aspergillosis, diabetes mellitus, and rheumatoid arthritis [22]. These differences in etiological distribution appear to be closely related to the socioeconomic development of countries and the prevalence of infectious diseases such as tuberculosis. While post-infectious and tuberculosis-related bronchiectasis remains prevalent in developing countries, immune-mediated and inflammatory causes are increasingly observed in developed regions.

Comorbidities were common in our cohort, with asthma, COPD, hypertension, psychiatric disorders, coronary artery disease, diabetes mellitus, and rheumatologic diseases being the most frequent. Previous studies have similarly demonstrated a high burden of comorbidities in non-cystic fibrosis bronchiectasis, particularly cardiovascular diseases, COPD, hypertension, asthma, and diabetes mellitus [23,24]. Variations among studies may be attributed to differences in age distribution, bronchiectasis etiology, and regional healthcare characteristics [24].

Radiological severity assessed using the Reid classification demonstrated a predominance of cystic bronchiectasis (71%) in our cohort, followed by varicose and tubular forms respectively. Comparable findings have been reported in other studies, particularly in cohorts including patients with advanced disease or those managed at tertiary referral centers [20,22,25]. The higher frequency of cystic bronchiectasis in our study may reflect referral bias and the inclusion of patients with more severe disease manifestations.

The distribution of affected lobes in presented cohort was consistent with previous reports, with predominant involvement of the lower lobes and the lingula. Similarly, pulmonary function test patterns observed in our patients were comparable to the literature, with obstructive impairment being the most frequent abnormality, followed by mixed and restrictive patterns. These findings support the representativeness of cohort despite differences in disease severity.

In the presented study, the coherence between the two scoring systems (FACED and BSI) were modest, consistent with earlier reports. FACED and BSI scores are widely used to assess disease severity and predict outcomes in bronchiectasis [26]. FACED was developed to predict 5-year mortality, while BSI predicts exacerbations, hospitalizations, lung function decline, and quality of life [26]. In this study, FACED classified the majority of patients as having mild disease, whereas BSI identified a higher proportion of patients with severe disease, consistent with previous studies. Previous investigations have reported variable coherence between FACED and BSI scores [27,28]. Differences in score composition, weighting of clinical parameters, and study populations may account for these discrepancies [29]. An important observation was the differential relationship between severity indices and inflammatory markers. While FACED scores were associated with IL-25 and IL-5 levels, BSI showed no significant association with cytokines. This finding suggests that FACED may better reflect biologically relevant disease activity, whereas BSI may be more influenced by clinical history and comorbid factors. This finding has potential clinical implications, suggesting that FACED may be more suitable for identifying biologically active disease phenotypes.

Importantly, no significant association was observed between BSI scores and alarmin or Th2 cytokine levels in our study. In contrast, FACED severity was significantly associated with increased IL-25 and IL-5 levels, suggesting that FACED may better reflect immunological pathways related to disease progression. IL-25, a member of the IL-17 cytokine family, is produced by epithelial cells, mast cells, macrophages, and hematopoietic progenitors, and acts as a key amplifier of Th2 inflammation by inducing IL-4, IL-5, and IL-13 production [30].

The divergent associations of FACED and BSI with epithelial alarmins may be explained by fundamental differences in the construction and clinical dimensions captured by these two severity indices. Although both scores quantify bronchiectasis severity, they are not interchangeable and assign different weights to structural, physiological, microbiological, and historical characteristics of the disease.

Experimental and clinical studies in asthma have demonstrated that elevated IL-25 levels are associated with enhanced Th2 inflammation, airway hyperresponsiveness, eosinophilia, and increased IgE levels [31]. Additionally, IL-25 has been implicated in airway remodeling and fibrosis through activation of structural cells [32]. Similar mechanisms may contribute to disease progression in bronchiectasis, particularly in patients with frequent exacerbations and severe disease.

Alarmins such as IL-25, IL-33, and TSLP have also been linked to viral and fungal infections, which are common triggers of exacerbations in bronchiectasis [33]. Increased IL-25 expression has been demonstrated in bronchial epithelial cells following rhinovirus infection, leading to enhanced Th2 inflammation [33]. In our cohort, the association between elevated IL-25 levels and disease severity may reflect underlying infectious burden, including undetected fungal colonization or chronic bacterial infection such as Pseudomonas aeruginosa.

IL-5 plays a central role in eosinophilic inflammation and contributes to airway hyperresponsiveness through its effects on smooth muscle cells [34]. Eosinophilic inflammation has been reported in up to one-third of patients with bronchiectasis, with elevated IL-5 and IL-13 levels observed in these patients [35]. Although anti–IL-5 therapies have primarily been studied in severe asthma, emerging evidence suggests potential benefits in patients with bronchiectasis overlapping with eosinophilic inflammation [36]. Recent literature strongly supports the therapeutic potential of targeting type 2 inflammation in specific bronchiectasis phenotypes. For instance, in a cohort of 300 patients, 7% were identified with an eosinophilic endotype (>300 cells/µL). When anti-IL-5 therapy was initiated in a severe subgroup of 15 patients—characterized by frequent exacerbations, high-dose corticosteroid dependence, and impaired quality of life—substantial clinical improvements were observed. Following a 6-month follow-up period, these patients demonstrated a marked reduction in both exacerbation frequency and mMRC dyspnea scores, underscoring the efficacy of biological agents in managing severe type 2-driven disease [37]. Our findings suggest that IL-25–driven Th2 inflammation may represent a treatable trait in bronchiectasis, particularly in patients with frequent exacerbations and severe disease. Targeting this pathway may help reduce disease progression and improve outcomes, highlighting the need for future prospective and interventional studies.

### Limitations

This study was conducted at a single tertiary care center, resulting in a relatively small sample size and potential selection bias toward more severe disease. Sputum cultures were evaluated semi-quantitatively, which may have limited pathogen detection. Additionally, cytokine and alarmin levels were measured at a single time point, preventing comparison between stable and exacerbation phases. Furthermore, a limitation of this study is the potential confounding effect of maintenance inhaled corticosteroid use in patients with asthma overlap, which may have partially suppressed Type 2 inflammatory markers and alarmin levels. From a clinical perspective, serum IL-25 has several potential advantages as a biomarker, including the use of a minimally invasive peripheral blood sample and the possibility of quantitative measurement using commercially available immunoassay-based methods. These characteristics could facilitate its integration into clinical practice if its prognostic value is confirmed. However, several barriers currently limit the routine use of IL-25 in bronchiectasis. Unlike established biomarkers such as peripheral blood eosinophil counts, serum IgE, or CRP, IL-25 measurement is not currently part of routine laboratory testing, standardized clinically validated cut-off values are lacking, and assay availability, inter-assay variability, sample processing requirements, and turnaround time may limit widespread implementation.

The presented study concluded that systemic inflammatory markers such as erythrocyte sedimentation rate, C-reactive protein, and neutrophil counts increased with disease severity. According to FACED classification, higher disease severity was associated with increased IL-25 and IL-5 levels. These findings suggest that alarmins and Th2-driven inflammation contribute to disease progression in non-cystic fibrosis bronchiectasis. In clinical practice, both BSI and FACED scores should be used together to assess mortality and exacerbation risk. Patients with frequent exacerbations, recent hospitalizations, and elevated IL-25 and IL-5 levels should be closely monitored due to their increased risk of adverse outcomes.

## 5. Conclusions

In patients with non-cystic fibrosis bronchiectasis, higher serum IL-25 levels were associated with greater disease severity and adverse clinical outcomes, including exacerbations and increased healthcare utilization. IL-5 levels were also associated with disease severity according to FACED classification. FACED and BSI showed modest agreement and differed in their associations with the evaluated inflammatory markers. These findings demonstrate an association between epithelial alarmin/type 2 inflammatory markers and clinical characteristics of bronchiectasis, particularly for IL-25, and warrant confirmation in larger prospective cohorts.

## Figures and Tables

**Figure 2:**
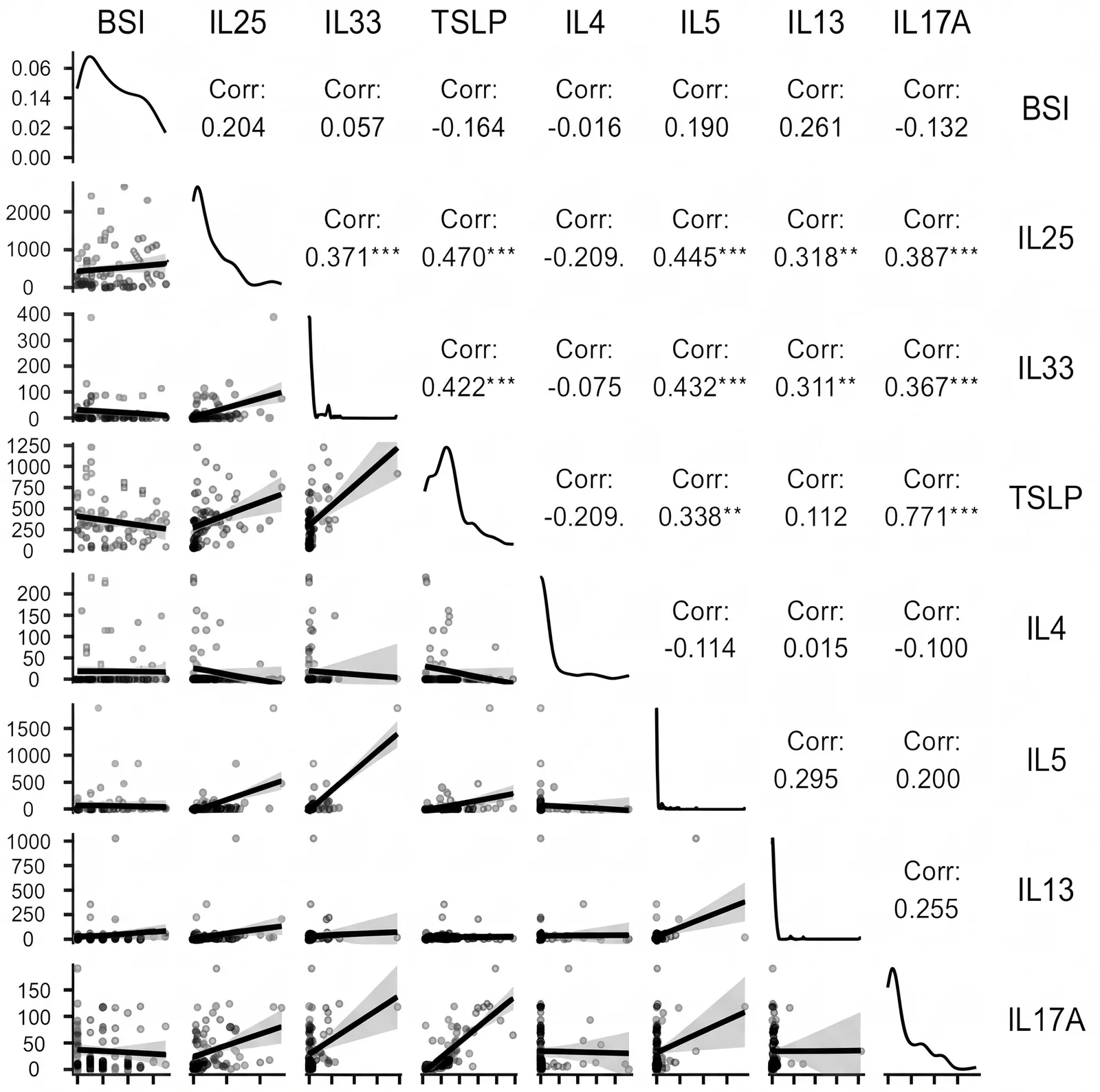
Correlation analysis between Bronchiectasis Severity Index (BSI) scores and interleukin levels. * *p* < 0.05, ** *p* < 0.01, *** *p* < 0.001.

**Table 1:** Sociodemographic characteristics and clinical profiles of the study population.

Variables	n or Mean ± SD (Min-Max)	(%)
**Age (years)**	50.19 ± 17.67	
**Gender**		
Female	44	55.70
Male	35	44.30
**Smoking Status**		
Smoker	22	27.80
Non-smoker	57	72.20
**Comorbidities**		
COPD	18	22.80
Asthma	27	34.20
Diabetes Mellitus	9	11.40
Hypertension	18	22.80
Coronary Artery Disease	13	16.50
Immunodeficiency	4	5.10
Rheumatological Disease	7	8.90
Psychiatric Disorders	14	17.70
Inflammatory Bowel Diseases	1	1.30
Sinusitis	6	7.60

COPD: Chronic obstructive pulmonary disease.

**Table 2:** Clinical and radiological characteristics of the patients.

Variables	n	(%)
**Etiology**		
Post-infectious	32	40.50
Prior Tuberculosis	6	7.60
COPD	18	22.80
Asthma	27	34.20
ABPA	3	3.80
Immunodeficiency	4	5.10
Rheumatological Disease	7	8.90
Primary Ciliary Dyskinesia	5	6.30
Inflammatory Bowel Diseases	1	1.30
Other	6	7.60
**Morphology**		
Tubular (Cylindrical)	11	14.50
Varicose	11	14.50
Cystic	57	71.00
**Number of Affected Lobes**		
1	10	12.70
2	28	35.40
3	15	19.00
4	7	8.90
5	19	24.10
**Lobe Involvement**		
Right Upper Lobe	49	62.00
Right Middle Lobe	37	46.80
Right Lower Lobe	45	57.00
Left Upper Lobe & Lingula	54	68.40
Left Lower Lobe	53	67.10
**Pulmonary Function Test (PFT)**		
Obstructive	30	38.00
Restrictive	9	11.40
Normal	26	32.90
Mixed Pattern	9	11.40

ABPA: Allergic Bronchopulmonary Aspergillosis, COPD: Chronic Obstructive Pulmonary Disease.

**Table 4:** Comparison of alarmin levels based on FACED scores.

Interleukin	Disease Severity (FACED)	n	Median [IQR]	*p*-Value
IL-25	Mild	54	210.93 [523.59] pg/mL	**0.016 **
	Moderate	15	953.03 [1057.39] pg/mL	
	Severe	9	330.95 [553.48] pg/mL	
IL33	Mild	54	1.87 [15.17] pg/mL	0.842
	Moderate	15	7.47 [14.83] pg/mL	
	Severe	9	5.14 [47.533] pg/mL	
TSLP	Mild	54	318.86 [263.92] pg/mL	0.916
	Moderate	15	255.57 [351.13] pg/mL	
	Severe	9	363.85 [321.43] pg/mL	
IL4	Mild	54	0[0] pg/mL	0.809
	Moderate	15	0[0] pg/mL	
	Severe	9	0 [16.74] pg/mL	
IL-5	Mild	54	0.5 [17.22] pg/mL	**0.031 **
	Moderate	15	9.01 [27.49] pg/mL	
	Severe	9	22.28 [58.97] pg/mL	
IL13	Mild	54	7.52 [22.29] pg/mL	0.097
	Moderate	15	19.44 [33.52] pg/mL	
	Severe	9	30.48 [59.41] pg/mL	
IL17A	Mild	54	15.18 [49.08] pg/mL	0.962
	Moderate	15	11.01 [48.71] pg/mL	
	Severe	9	14.09 [71.7] pg/mL	

IQR: Interquartile Range. Bold typeface has been used for statistically significant findings.

**Table 5:** Agreement and Correlation Between BSI and FACED Severity Scores.

FACED			BSI		Total
		Mild	Moderate	Severe	
	Mild	30	12	12	54
	Moderate	0	4	12	16
	Severe	1	0	8	9
Total		31	16	32	79
Statistical Method			Cohen’s Kappa		
Percentage of Agreement (%)			58		
Cohen’s Kappa Coefficient (ĸ)			0.273		
*p*-value			<0.001		

**Table 6:** Logistic regression analysis for FACED score.

FACED	Parameters	B	Standard Error	*p*	OR	95% IR	
						Lower limit	Upper limit
1	Hospitalization	1.788	0.438	**<0.001 **	5.976	2.534	14.097
2	IL-25	0.001	0	**0.009**	1.001	1	1.002
	Hospitalization	1.996	0.5	**<0.001 **	7.357	2.761	19.604
3	IL-25	0.002	0.001	**0.005**	1.002	1	1.003
	ER admission	0.785	0.309	**0.011**	2.193	1.197	4.02
	Hospitalization	1.654	0.528	**0.002**	5.226	1.856	14.715

ER: Emergency room IR: Confidence İnterval. Bold typeface is used for statistically significant data.

## Data Availability

The datasets generated and/or analysed during the current study are deposited into the Figshare database under the DOI/accession number 10.6084/m9.figshare.32536893 and are available at the following URL: https://doi.org/10.6084/m9.figshare.32536893.
